# A highly conserved redox-active Mx_(2)_CWx_(6)_R motif regulates Zap70 stability and activity

**DOI:** 10.18632/oncotarget.16486

**Published:** 2017-03-22

**Authors:** Christoph Thurm, Mateusz P. Poltorak, Elisa Reimer, Melanie M. Brinkmann, Lars Leichert, Burkhart Schraven, Luca Simeoni

**Affiliations:** ^1^ Institute of Molecular and Clinical Immunology, Health Campus Immunology, Infectiology, and Inflammation, Otto von Guericke University, Magdeburg, Germany; ^2^ Viral Immune Modulation Group, Helmholtz Centre for Infection Research, Braunschweig, Germany; ^3^ Institute for Biochemistry and Pathobiochemistry, Ruhr University, Bochum, Germany; ^4^ Department of Immune Control, Helmholtz Centre for Infection Research, Braunschweig, Germany; ^5^ Current address: Institut fr Medizinische Mikrobiologie, Immunologie und Hygiene, Technische Universität München, Munich, Germany

**Keywords:** Zap70, oxidation, TCR signaling, Cdc37, protein stability, Immunology and Microbiology Section, Immune response, Immunity

## Abstract

ζ-associated protein of 70 kDa (Zap70) is crucial for T-cell receptor (TCR) signaling. Loss of Zap70 in both humans and mice results in severe immunodeficiency. On the other hand, the expression of Zap70 in B-cell malignancies correlates with the severity of the disease. Because of its role in immune-related disorders, Zap70 has become a therapeutic target for the treatment of human diseases. It is well-established that the activity/expression of Zap70 is regulated by post-translational modifications of crucial amino acids including the phosphorylation of tyrosines and the ubiquitination of lysines. Here, we have investigated whether also oxidation of cysteine residues regulates Zap70 functions. We have identified C575 as a major sulfenylation site of Zap70. A C575A substitution results in protein instability, reduced activity, and increased dependency on the Hsp90/Cdc37 chaperone system. Indeed, Cdc37 overexpression reconstituted partially the expression but fully the function of Zap70C575A. C575 lies within a Mx_(2)_CWx_(6)_R motif which is highly conserved among almost all human tyrosine kinases. Mutation of any of the conserved amino acids, but not of a non-conserved residue preceding the cysteine, also results in Zap70 instability. Collectively, we have identified a new redox-active motif which is crucial for the regulation of Zap70 stability/activity. We believe that this motif has the potential to become a novel target for the development of therapeutic tools to modulate the expression/activity of kinases.

## INTRODUCTION

Signaling *via* the TCR (T-cell receptor) plays an essential role in the regulation of T-cell differentiation, survival and proliferation. All these processes are crucial for the adaptive immune response against invading pathogens and hence they are tightly controlled at the molecular level. Indeed, alterations in the expression or in the activation of signal-transducing mediators are at the basis of dysregulated immune responses such as autoimmunity and of other human diseases including immunodeficiency and cancer [1].

The TCR is a multi-protein complex including an αβTCR heterodimer (or γδTCR in a minor subset of T cells) and also CD3γδε- and ζ-chains. The CD3/ζ-chains contain ITAMs (immunoreceptor tyrosine-based activation motifs), which are required for signaling [2]. Upon antigen recognition by the TCR/CD3/ζ complex, a hierarchical signaling cascade is initiated which includes: (i) phosphorylation of the ITAMs on key tyrosine residues by the tyrosine kinase Lck (ii) recruitment of the cytosolic Syk-family tyrosine kinase Zap70 (ζ-chain-associated protein of 70 kDa) to the phosphorylated ITAMs, (iii) phosphorylation and activation of Zap70 by Lck, (iv) phosphorylation of the transmembrane adaptor protein LAT and of others effector molecules [3]. These events will lead to the activation of intracellular signaling pathways such as Ras-MAPK, PKCs, and Ca^2+^ flux, thus culminating in transcriptional activation and cell responses.

Zap70 is one of the crucial molecules involved in the initiation of TCR signaling. Its importance has been demonstrated by the observation that Zap70-deficiency in humans results in a Severe Combined Immunodeficiency (SCID) characterized by the absence of CD8^+^ T cells and in a defective activation of the CD4^+^ T-cell compartment [4–6]. Also in mice, loss of Zap70 results in a profound developmental block at the DP stage in the thymus [7, 8]. Most of the Zap70 mutations found in SCID patients result in protein instability [4, 5, 9, 10]. Recently, new Zap70 mutations, which do not result in immunodeficiency but rather in severe autoimmunity, have been described [11]. Finally, Zap70 plays also an important role in the pathology of many B cell-derived malignancies [12, 13].

Because of its involvement in both physiological processes and pathological modifications, the regulation of Zap70 activity has been extensively studied. Zap70 possesses two tandem SH2 domains and a carboxy-terminal kinase domain [14]. Two linker regions, defined as interdomain A (I-A) and interdomain B (I-B), separate the two SH2 domains and the SH2 domain from the kinase domain, respectively. In resting T cells, Zap70 is located in the cytosol in an auto-inhibited conformation. The recruitment of Zap70 to the phosphorylated ITAMs *via* its tandem SH2 domains perturbs the auto-inhibited conformation and favors the phosphorylation of Y315 and Y319 in the interdomain B, thus leading to the stabilization of the active enzyme [15–18].

Reversible phosphorylation on tyrosine residues is crucial for the regulation of Zap70 activity [17]. For example, phosphorylation of Y315 and Y319 in the interdomain B stabilizes the active enzyme and additionally provides docking sites for other effector molecules including Vav and Lck, respectively. Conversely, Y292 serves as docking site for c-Cbl (casitas B-lineage lymphoma) and plays an inhibitory role in TCR signaling likely by mediating Zap70 ubiquitination and degradation. Two additional tyrosines located in the kinase domain have also been implicated in the regulation of the kinase activity. Whereas phosphorylation of Y492 inhibits Zap70 activity, phosphorylation of Y493 results in full kinase activation.

In addition to phosphorylation, other reversible post-translational modifications of Zap70 have been described which are also involved in the regulation of its activity. Ubiquitination of K578 by Nrdp1 in CD8^+^ T cells has been shown to inactivate Zap70 by favoring Sts1- and Sts2-mediated Zap70 dephosphorylation [19]. More recently, additional ubiquitination sites in Zap70 have been identified [20]. One of these sites, K217, appears to be a novel negative regulator of Zap70 function and TCR signaling. However, the molecular mechanisms of how ubiquitination of K217 inhibits Zap70 remain still elusive.

Also cysteine residues have recently become the focus of intensive investigation, as they function as “switches” upon oxidation of the thiol group and hence may represent an additional mechanism to regulate protein function [21].

During the last years, evidence has been provided that modification of the thiol (-SH) to sulfenic acid (-S-OH) in the cysteine residues of tyrosine kinases appears to be involved in regulating their activity [22]. Very little is known about redox-dependent regulation of Zap70 functions. Recently, it has been shown that sulfenylation of C39 located in the phosphotyrosine-binding pocket of Zap70 SH2 domain prevents the binding of the tandem SH2 domains to the phosphorylated ITAMs [23]. To our knowledge, this is the only described example of a redox-active cysteine which is crucial for the regulation of Zap70 activation.

In this study, we have identified a novel redox-active cysteine located within a highly conserved amino acid sequence which is involved in the regulation of Zap70 stability and function.

## RESULTS AND DISCUSSION

### A redox-active Mx(2)CWx(6)R motif regulates the stability and activity of Zap70

Cysteines have been proposed to function as redox switches regulating the activity of protein tyrosine kinases (PTKs). For example, early studies on Src showed that two cysteines at position 245 and 487, which were demonstrated to form an intramolecular disulfide bridge, regulate its kinase activity [24]. Besides Src, a number of kinases were shown to be regulated in a redox dependent manner including c-Abl and AKT [25, 26]. In response to reactive oxygen species (ROS), c-Abl forms mixed disulfides and thereby becomes inactive [25]. Conversely, AKT becomes activated by oxidation upon angiotensin treatment in smooth muscle cells [26].

**Figure 1:**
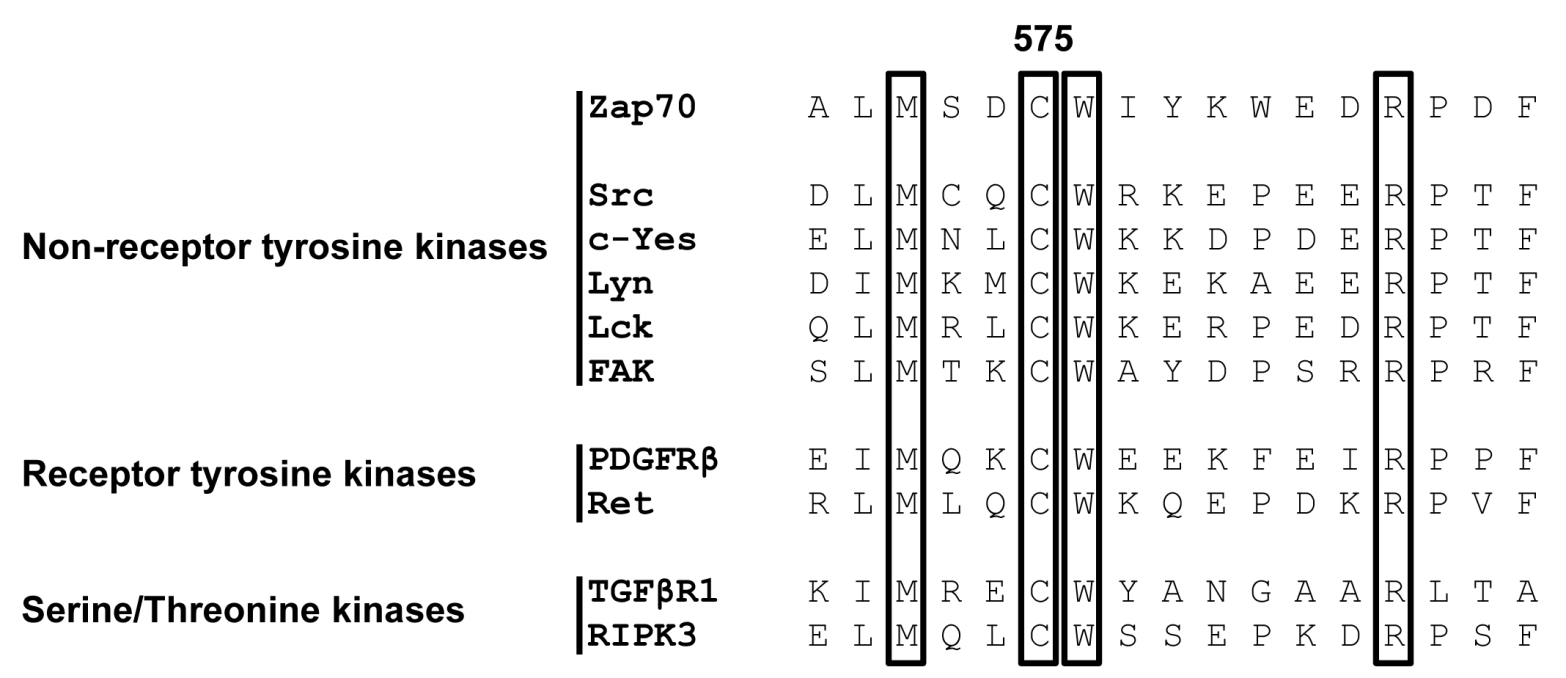
Alignment of the primary amino acid sequence of the conserved Mx _(2)_CWx_(6)_R motif in non-receptor and receptor tyrosine kinases, as well as serine/threonine kinases The number 575 indicates the position of the core cysteine in Zap70.

**Figure 2:**
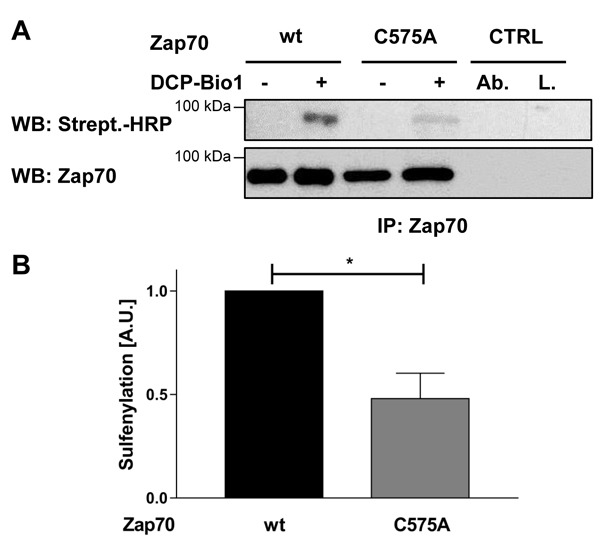
Sulfenylation of Zap70wt and the C575A mutant in P116 cells P116 cells were reconstituted with either Zap70wt or the C575A mutant and treated with either DCP-Bio1 (+) or DMSO (-) for 1h. Subsequently, cells were lysed and Zap70 was immunoprecipitated using a specific antibody. Proteins were separated using SDS-PAGE and sulfenylated Zap70 was detected using Stretpavidin-HRP. Membranes were probed against total Zap70 to show equal loading. The amount of the loaded wt sample was reduced to normalize the level of wt Zap70 to that of the C575A mutant, as described in the material and methods section. **A**. Representative western blot for Zap70 sulfenylation. **B**. Densitrometric analysis of Zap70 sulfenylation normalized to total Zap70 (*n* = 3). Ctrl, immunoprecipitation controls. Ab., beads were incubated with immunoprecipitating antibody and lysis buffer alone. L., beads were incubated with lysates alone without immunoprecipitating antibody.

**Figure 3:**
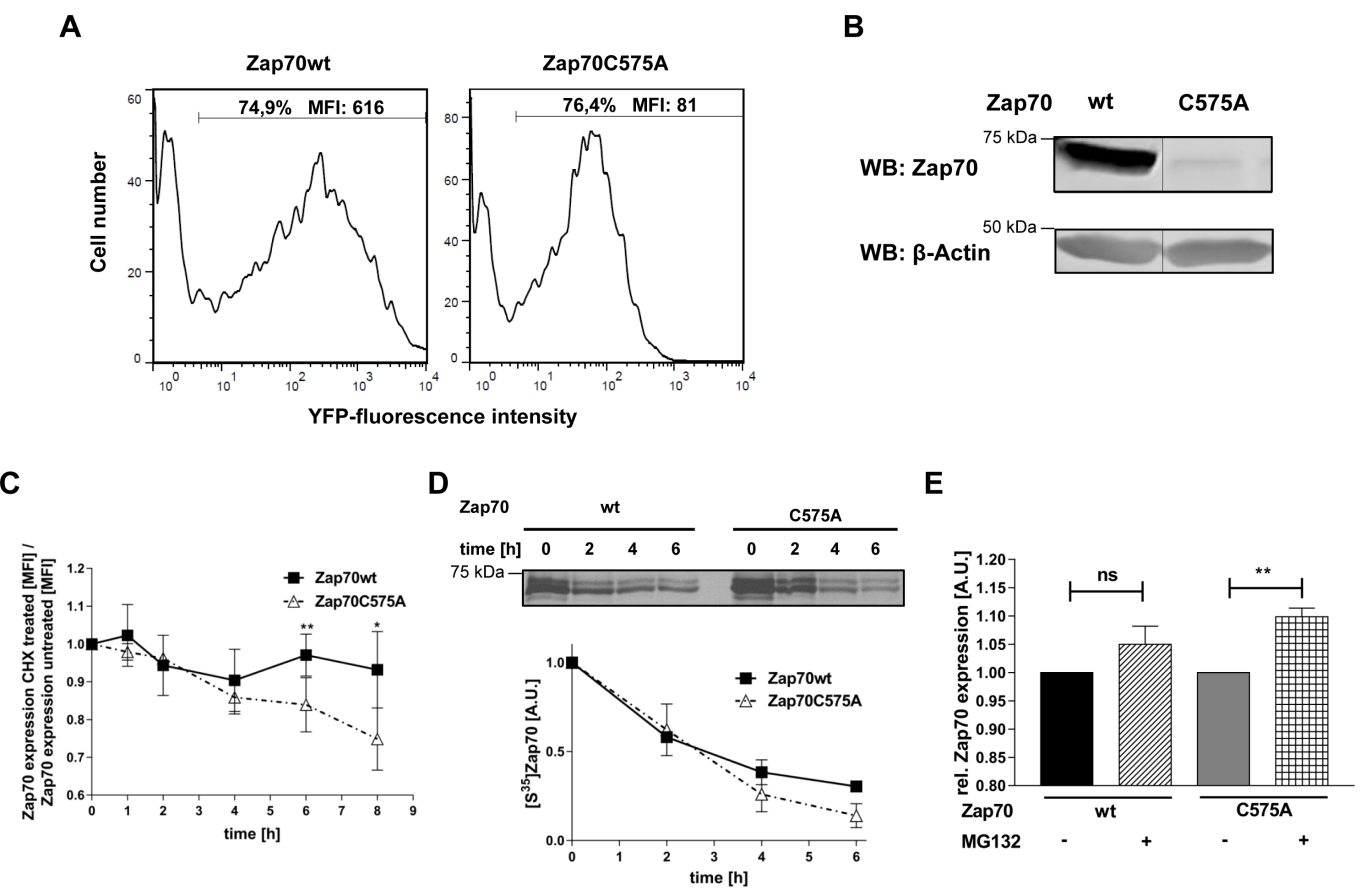
Analysis of protein stability of Zap70C575A **A**. Representative histograms of the expression of Zap70 wt and C575A. P116 cells were transfected with YFP-tagged Zap70wt or C575A. Transfection efficiency was determined by gating on YFP^+^ cells. **B**. Zap70wt or C575A expressing P116 cells were lysed and subjected to immunoblot analysis with an antibody against human Zap70. **C**. P116 cells reconstituted with either Zap70wt-YFP or C575A-YFP were either left untreated or treated with 20μg/mL cycloheximide for different time points up to 8h. Zap70 protein expression was measured by flow cytometry. The values of wt and mutant Zap70-YFP MFI were normalized to the respective untreated control samples for each time point followed by normalization to the 0h time point which was set to 1 for both wt and mutant Zap70 (*n* = 3). **D**. HEK293T cells were transfected with Zap70-Myc-DDK or C575A-Myc-DDK. After starvation of cysteine and methionine, cells were pulsed with [S35]methionine/cysteine for 15min, followed by chase up to 6h. Subsequently, cells were lysed and Zap70 was immunoprecipitated using anti-FLAG antibody. Samples were separated using SDS-PAGE and signals were detected by autoradiography and quantified by densitometry (*n* = 2). **E**. Zap70wt-YFP of C575A-YFP expressing P116 cells were either treated with 20mM MG132 (+MG132) or DMSO (-MG132) for 4h. Subsequently, protein expression was measured by flow cytometry. YFP MFI values of wt and mutant Zap70 were normalized to the respective untreated control samples which were set to 1 (*n* = 4).

Sequence alignment analysis revealed that many receptor and non-receptor PTKs, as well as serine/threonine kinases, share a highly conserved amino acid Mx_(2)_CWx_(6)_R motif in which a cysteine is one of the conserved amino acids (Figure 1). This motif has been proposed to be involved in the redox-dependent regulation of kinase activity and stability [27]. However, no data demonstrating the functional importance of this motif have been generated so far. The tyrosine kinase Zap70 also possesses this motif. We initially assessed whether the cysteine in the consensus sequence is oxidized using a cell permeable probe (DCP-Bio1) to trap sulfenylated cysteines [28]. We expressed Zap70wt or a Zap70 mutant carrying a C to A mutation (Zap70C575A) in P116, a Zap70-deficient Jurkat T-cell variant [29]. Upon treatment with DCP-Bio1, Zap70 was immunoprecipitated and global cysteine sulfenylation was assessed by immunoblotting using HRP-conjugated streptavidin. Figure 2A indicates that Zap70wt is clearly sulfenylated in P116 cells. Quantifications of the sulfenylation of Zap70C575A revealed a 50% reduction of total sulfenylation, thus indicating that C575 is a major target of sulfenylation in Zap70 (Figure 2A, 2B). We have also found that Zap70 is sulfenylated in a similar fashion in both resting and CD3-stimulated primary human T cells (data not shown). We next assessed whether oxidation of C575 is required for the regulation of Zap70 function. Previously published data revealed that substitution of the corresponding cysteine in Lck (C475) and Src (C498) with alanine resulted in a strong reduction of the half-life of the protein [30, 31]. Therefore, we next tested whether the C575A mutation also affects Zap70 stability. We initially expressed a Zap70 YFP-tagged wt and C575A mutated constructs and assessed YFP expression by flow cytometry. The data depicted in Figure 3A clearly show that both constructs are transfected with the same efficiency, as the proportion of YFP^+^ cells are comparable between wt and mutant Zap70 samples. However, the YFP MFI are markedly lower in cells expressing Zap70C575A, thus indicating that the mutant protein is expressed at lower levels. We next corroborated these results by expressing myc-tagged wt or Zap70C575A in P116 cells. The expression of wt Zap70 was detectable by western blot at the expected size. However, the protein level of the C575A mutant was strongly reduced (about 95%) compared to wt (Figure 3B). Similar results were obtained upon expression of Zap70 constructs in HEK293T cells (data not shown). Thus, these data indicate that Zap70C575A is unstable and degraded.

In order to further analyze the stability of the Zap70 mutant, cells were treated with cycloheximide, an inhibitor of *de novo* protein synthesis, for different time points and subsequently Zap70 expression levels were normalized to the respective untreated control. Upon cycloheximide treatment, the expression of Zap70wt stayed constant for all tested time points, thus indicating that Zap70 has a long turnover and is stable. In contrast, cycloheximide treatment led to a progressive reduction of Zap70C575A, whose expression was significantly reduced 6h after treatment (Figure 3C).

To further evaluate the stability of Zap70C575A, we conducted pulse-chase experiments (Figure 3D). Wt or mutant Zap70 expressing HEK cells were labeled with S^35^methionine and cysteine followed by a chase up to 6h. Labeling of Zap70C575A was strongly reduced to about 50% compared to Zap70wt after 4 and 6h. These data suggest that the introduction of the C to A substitution at position 575 reduced protein stability.

To examine the degradation mechanism of Zap70C575A, we used different inhibitors. Inhibition of proteasomal degradation using MG132 had no effect on Zap70wt, but significantly increased the protein level of Zap70C575A (Figure 3E). Different inhibitors of proteases or the inhibition of lysosomal degradation did not affect the stability of Zap70C575A, thus suggesting that mutant Zap70 is degraded, at least in part, *via* the proteasome.

**Figure 4:**
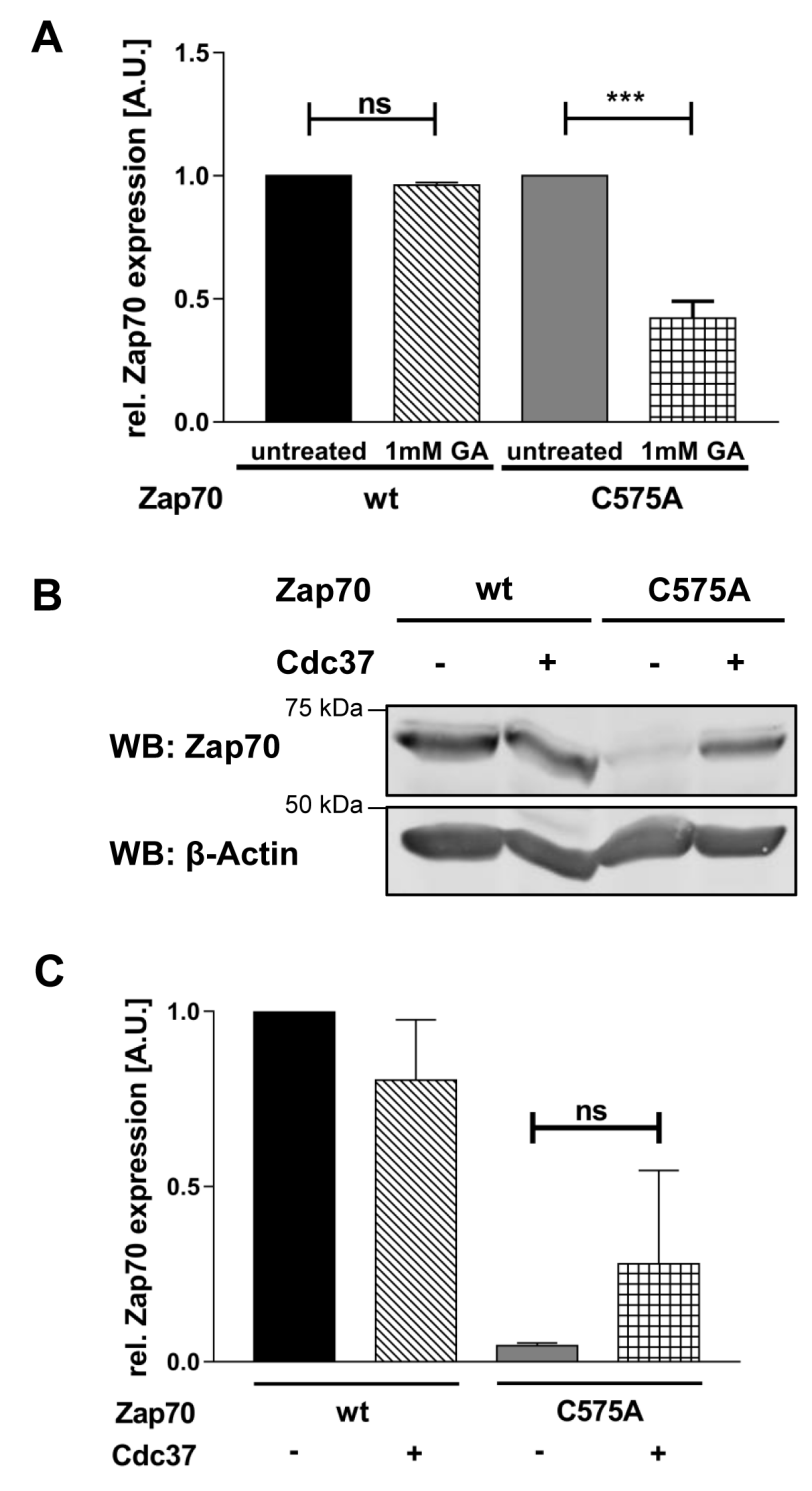
The Hsp90/Cdc37 complex stabilizes the expression of Zap70C575A **A.** For inhibition of Hsp90, P116 cells were reconstituted with Zap70wt-YFP or C575A-YFP and either left untreated or treated with 1mM geldanamycin for 4h. Zap70-YFP expression was measured by flow cytometry and YFP MFI were normalized to untreated control which was set to 1 for both wt and mutant Zap70 (*n* = 3). **B**. P116 cells were transfected with Zap70-Myc-DDK and Cdc37-Myc-DDK. Cells were lysed and Zap70 protein expression was analyzed using SDS-PAGE and western blot. β-actin was used as loading control. **C**. Quantification of Zap70 expression. Intensity of the bands of all samples were normalized to β-actin followed by an additional normalization to the wt sample without Cdc37 overexpression which was set to 1 (*n* = 4).

Chaperones are crucial for the regulation of protein stability. In particular, Hsp90 in conjunction with Cdc37 are known to be important for the stabilization of various PTKs [32]. Therefore, we investigated if the C575A mutation alters the interplay with these chaperons. Figure 4A clearly shows that the expression of mutant Zap70 strongly depends on Hsp90α, as inhibition of Hsp90α further destabilizes mutant but not wt Zap70. As Cdc37 appears to be a co-chaperone specifically regulating the activity and stability of kinases, we tested if the overexpression of this co-chaperone further stabilizes Zap70C575A. We have found that the overexpression of Cdc37 partially stabilizes the expression of Zap70C575A, but not Zap70wt (Figure 4B and 4C).

**Figure 5:**
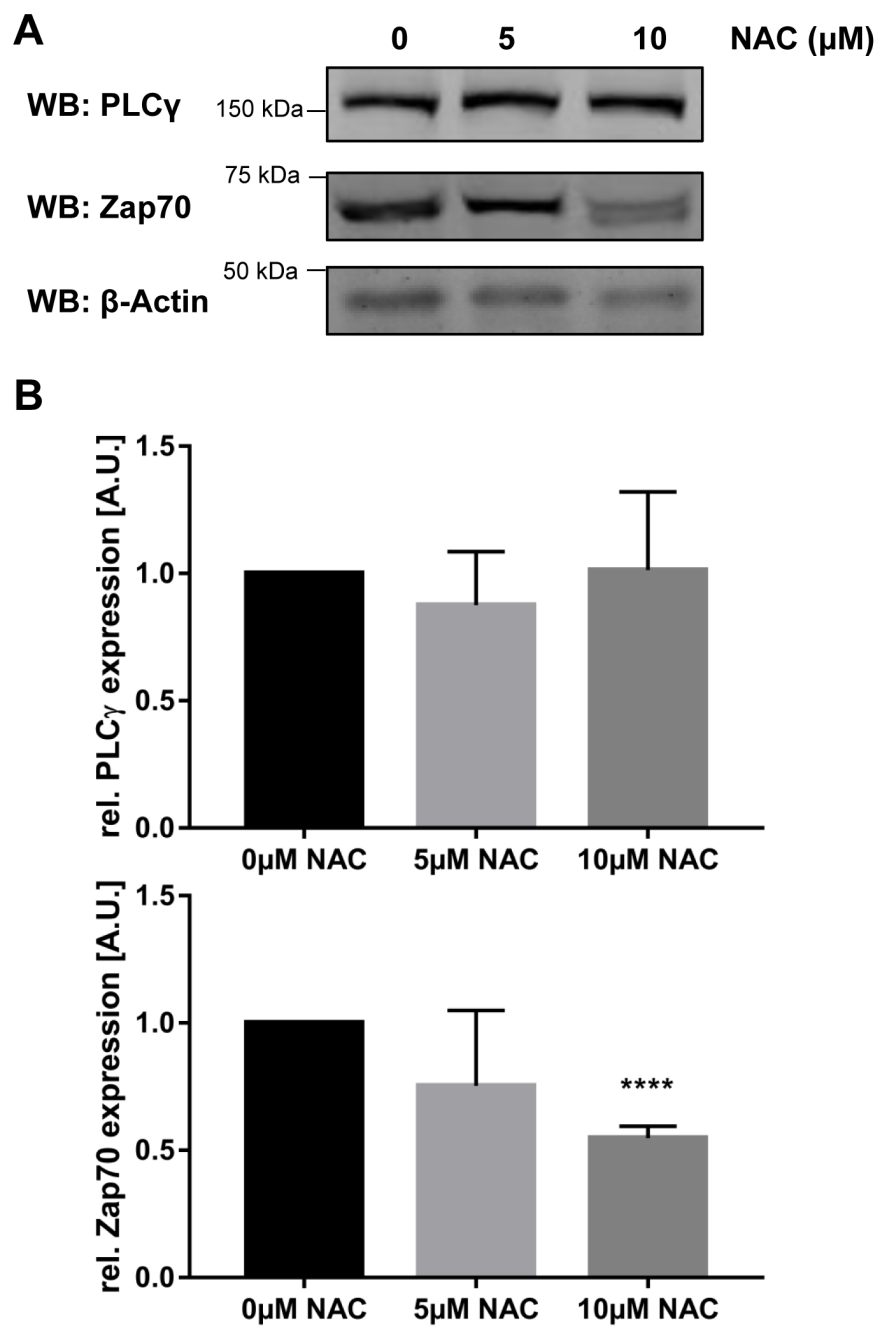
Treatment with NAC destabilizes Zap70wt Jurkat T cells were either left untreated or treated with 5μM or 10 μM N-acetylcysteine (NAC) for 96h. Cells were lysed and subjected to immunoblot analysis using the indicated antibodies. **A**. Representative western blots. **B**. Quantification of PLCγ and Zap70 expression (*n* = 3).

If oxidation is important for the regulation of Zap70 stability, we thought that treatment with antioxidants should result in a reduction of Zap70 expression. To test this hypothesis, we treated Jurkat T cells with N-acetylcysteine (NAC) and assessed Zap70 expression by western blotting. As shown in Figure 5, the expression of Zap70 was significantly decreased upon treatment with 10μM NAC. Importantly, the expression of PLCγ, which does not possess the conserved C575, is not affected upon treatment with antioxidant (Figure 5). In summary, these data indicate that the C575 located in the Mx_(2)_CWx_(6)_R motif represents a new redox-active residue regulating Zap70 stability.

**Figure 6:**
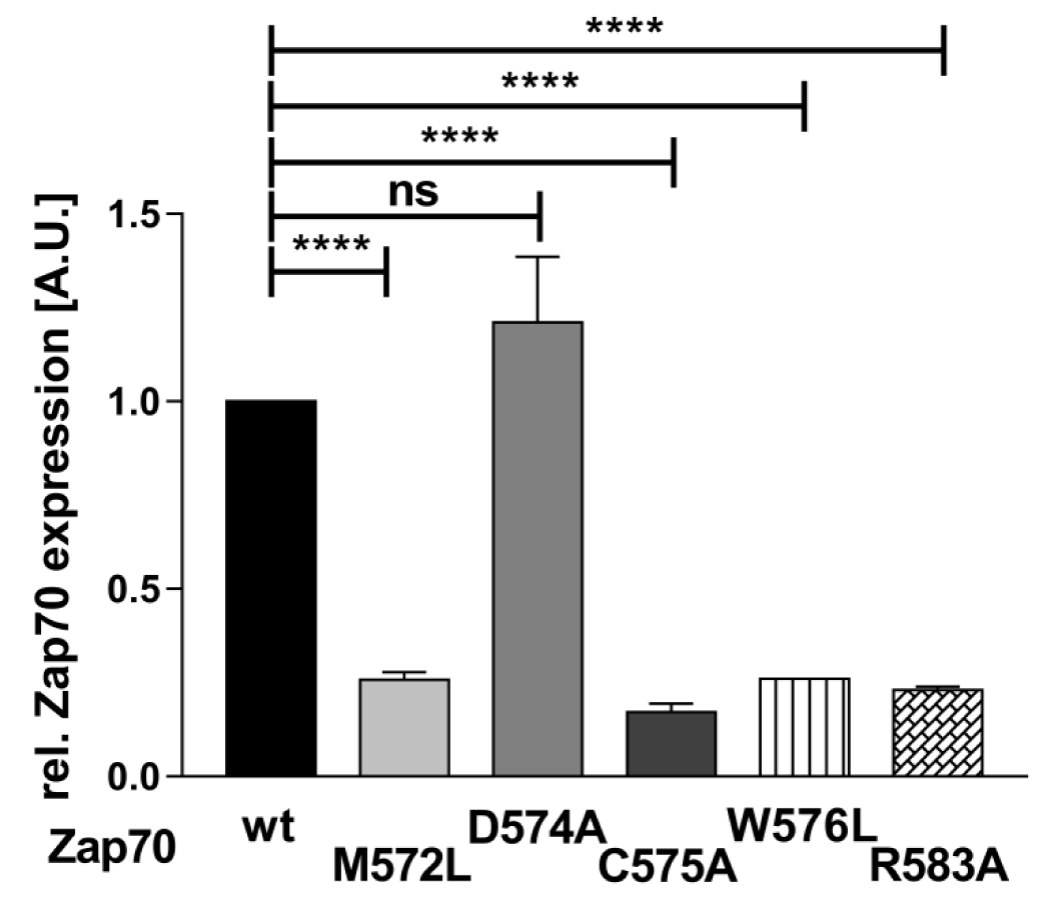
Expression of Zap70 is regulated by the Mx _(2)_CWx_(6)_R motif To assess the role of the conserved amino acids within the Mx_(2)_CWx_(6)_R motif, YFP-tagged Zap70wt or the respective point mutants were expressed in P116 cells. Protein expression was analyzed by flow cytometry. YFP MFI values of all mutants were normalized to the MFI of Zap70wt which was set to 1 (*n* = 3).

By using the MOTIF search of the GenomeNet database (www.genome.jp), we have found that about 99 human PTKs and serine/threonine kinases possess this Mx_(2)_CWx_(6)_R consensus sequence (Figure 1). Interestingly, a previous study has shown that a M572L mutation in humans results in the loss of Zap70 expression and may be the cause of immunodeficiency [10]. Therefore, we next assessed whether also the other conserved amino acid are required for the regulation of Zap70 stability. We mutated the conserved amino acids to alanine or leucine and Zap70 expression was assessed. Figure 6 clearly indicates that M572L, W576L, and R583A Zap70 mutants show a decreased expression. Conversely, the mutation of the non-conserved aspartate at position 574 (just preceding the cysteine) to alanine does not affect Zap70 stability. Collectively, all conserved amino acids in the Mx_(2)_CWx_(6)_R motif appear to be crucial for the stability of Zap70.

### Zap70C575A shows defective TCR-mediated signaling

**Figure 7:**
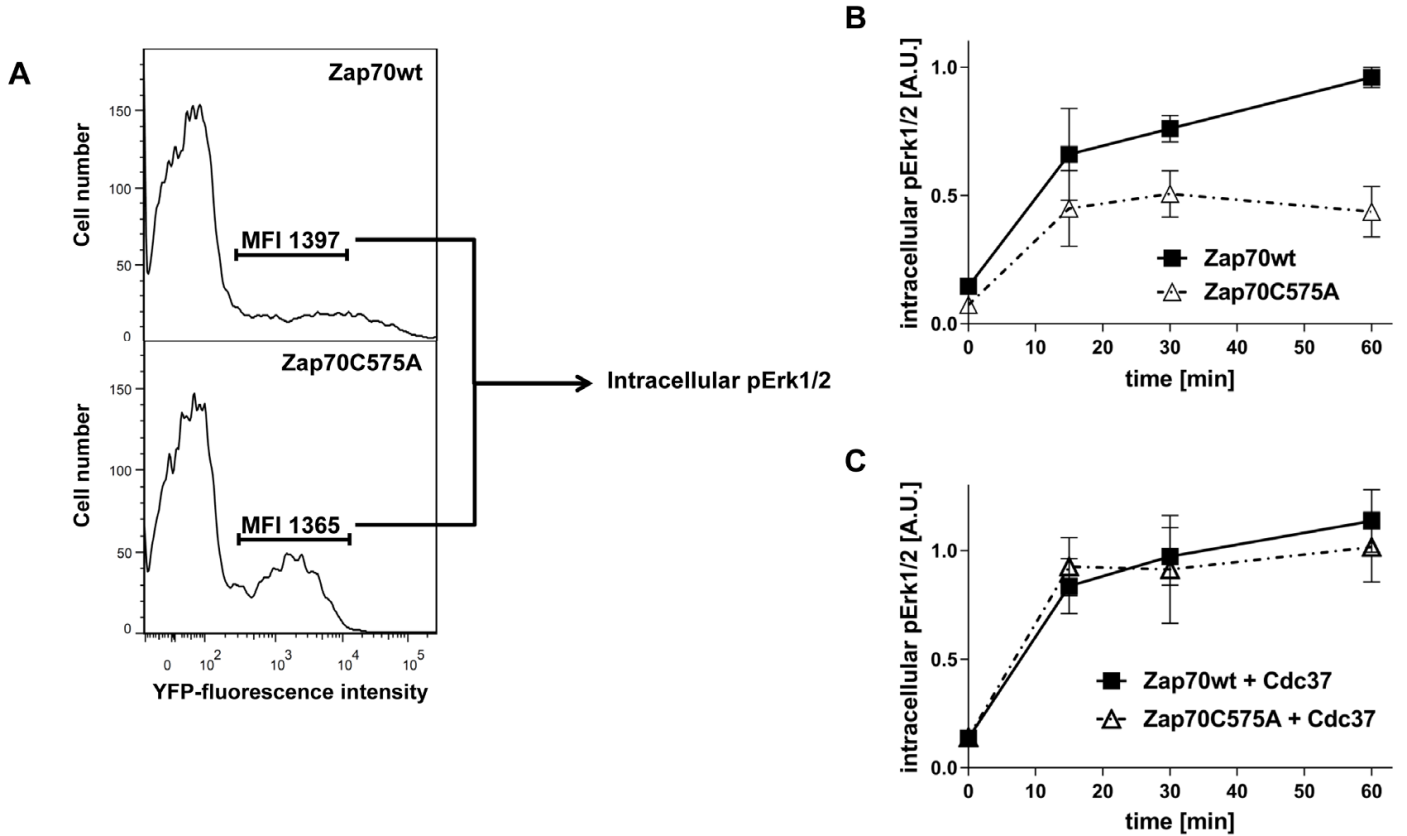
Analysis of Zap70 function P116 cells were transiently transfected with Zap70wt-YFP or Zap70C575A-YFP. **A**. Gating strategy for the evaluation of TCR signaling. Cells were gated for similar mean fluorescence intensity (MFI) of the YFP-tag. **B**. To evaluate the function of Zap70C575A on TCR-mediated Erk1/2 activation, P116 cells were transfected with Zap70wt-YFP or C575A-YFP. Cells were stimulated with anti-CD3 antibodies (UCHT1) immobilized on microspheres for different time points. Cells displaying similar YFP fluorescence intensity were analysed for the level of intracellular phosphorylated Erk1/2 (*n* = 3). **C**. To evaluate the effect of Cdc37 on the function of Zap70C575A, P116 cells were transfected with Zap70wt-YFP or C575A-YFP upon Cdc37 overexpression and stimulated as described above. Cells displaying similar YFP fluorescence intensity were analysed for the level of intracellular phosphorylated Erk1/2 (*n* = 3).

The cysteine within the Mx_(2)_CWx_(6)_R motif has also been proposed to be required for the regulation of kinase activity [27]. Because of the strong reduction in the expression of Zap70C575A, a deep characterization of the mutant molecule by standard immune blots was not possible. Therefore, we took advantage of the YFP-tagged Zap70 for signaling studies using intracellular staining and flow cytometry. Wt Zap70-YFP or C575A Zap70-YFP were expressed in P116 cells. We next evaluated the ability of the constructs to reconstitute TCR-mediated Erk1/2 phosphorylation using intracellular flow cytometry by gating on cells displaying comparable MFI and hence expressing similar amount of Zap70 (Figure 7A). This analysis revealed that Zap70C575A displayed a strong defect in the reconstitution of TCR-mediated Erk1/2 activation compared to the wt protein (Figure 7B).

Cdc37 is also known to regulate kinase activity [33]. Therefore, we investigated whether Cdc37 overexpression, in addition to stabilizing Zap70C575A expression, also affects Zap70C575A function. As shown in Figure 7C, Cdc37 completely reconstituted Erk1/2 phosphorylation. Thus, Cdc37 appears to be a crucial factor for the stability and more importantly for the activity of Zap70C575A.

## CONCLUSIONS

Herein, we have identified a redox-active highly conserved amino acid Mx_(2)_CWx_(6)_R motif located at the C-end of the kinase domain of Zap70 which is crucial for the regulation of Zap70 stability/activity. The oxidation of the cysteine within this motif appears to be crucial not only for Zap70, as its substitution in Src (C498A), Yes (C506A), Lyn (C479A), Lck (C475A), c-Ret (C376A), PDGFR-β (C940A), and FAK (C685A) affects kinase activity and in some cases also protein stability [24, 30, 31, 34–39]. We have shown for the first time that in addition to the cysteine also the other conserved amino acids are crucial for protein stability. As all the conserved residues face the same cavity of the kinase lobe, it is likely that they all act in concert to coordinate the oxidation of the cysteine.

These findings suggest that treatment with antioxidants could be beneficial for the therapy of human diseases in which kinases are overexpressed or hyperactive. Of particular interest are chronic B-cell leukemia (BCLL) where Zap70 expression correlates with a more aggressive behaviour of BCLL and disease progression [12, 13]. Indeed our study additionally suggests that treatment with antioxidants reduces Zap70 expression and hence it might be beneficial for the treatment of BCLL in combination with standard clinical protocols involving kinase inhibitors. We believe that further structure/functional analysis of this motif combined with in-depth biochemical studies will provide the basis for the development of new therapeutic tools to modulate the expression/activity of Zap70 and possibly other kinases.

## MATERIALS AND METHODS

### Cells and materials

HEK-293T cells (human embryonic kidney 293T cells) were cultured in DMEM (Dulbecco's modified Eagle's medium) with 10% (v/v) FBS (fetal bovine serum). JE6 and P116 cells were cultured in RPMI 1640 (Roswell Park Memorial Institute) with 10% (v/v) FBS in the presence or absence of inhibitors. Anti-Myc (9E10), anti-Zap70 (1E7.2), and ProteinG Plus agarose were purchased from SantaCruz Biotechnology. Anti-FLAG and anti-phospho 44/42 MAPK were obtained from Cell Signaling Technology and biotinylated anti-CD3ε (UCHT1) from BioLegend. Anti-β-Actin antibody, MG132, N-acetylcysteine and Geldanamycin were purchased from SigmaAldrich and Cycloheximide from Carl Roth. DCP-Bio1 was from MerckMillipore, Streptavidin-HRP, was from ThermoFischer, [S35]Methionine/Cysteine was from Hartmann Analytic GmbH and the goat-anti rabbit APC was from Jackson ImmunoResearch.

### Site directed mutagenesis

For site directed mutagenesis of Zap70 the Agilent Quick Change II XL (Agilent) system was used according to the manufacturer's instructions. The plasmids pEYFP-N1 Zap70 and pCMV6-Entry Zap70-Myc-DDK were used as templates and primers were designed using the tools from Agilent and synthesized by Biomers (Biomers.net GmbH). The following primers were used:

Zap70M572L

FWD:  c gaa ctg tac gca ctc ttg agt gac tgc tgg at

REV:  at cca gca gtc act caa gag tgc gta cag ttc g

Zap70D574A

FWD:  g tac gca ctc atg agt gcc tgc tgg atc tac aag t

REV:  a ctt gta gat cca gca ggc act cat gag tgc gta c

Zap70C575A

FWD:  ac gca ctc atg agt gac gcc tgg atc tac aag tgg g

REV:  c cca ctt gta gat cca ggc gtc act cat gag tgc tg

Zap70W576L

FWD:  a ctc atg agt gac tgc ttg atc tac aag tgg gag g

REV:  c ctc cca ctt gta gat caa gca gtc act cat gag t

Zap70R583A

FWD:  tgg atc tac aag tgg gag gat gcc ccc gac ttc c

REV:  g gaa gtc ggg ggc atc ctc cca ctt gta gat cca

Mutations were confirmed by sequencing.

### Transfection by calcium phosphate

HEK cells were seeded onto 6-well tissue culture plates and transfected at approximately 60% confluence. 300μL of 250mM CaCl supplemented with 6μg cDNA were added dropwise under constant agitation to 300μL 2xHBS buffer (280mM NaCl, 10mM KCl, 1,5mM Na2HPO4 × 2 H2O, 12mM Glucose und 50mM HEPES). After incubation for 25min at RT, 3mL of DMEM/10%FCS was added and the mixture was carefully pipetted into the well. Cells were incubated in the presence of transfection medium for 24h.

### Transfection by electroporation

DNA electroporation was performed with the Gene Pulser II System (BIORAD, Hercules, CA, USA). P116 cells were cultured as described above. 2 × 10^7^ cells were resuspended in 350μL RPMI/10%FCS and transferred together with 5-30 μg of cDNA into a 4 mm electroporation cuvette (VWR). Electroporations were carried out with 230V and 950μF. Subsequently, cells were transferred in 50 mL pre-warmed RPMI/10%FCS (25mL fresh/25mL conditioned) and cultured for 12-16h.

### DCP-bio1 labeling

P116 cells were transfected with 30μg Zap70-YFP plasmids and incubated over night. Subsequently, transfected P116 cells were incubated with 1mM DCP-Bio1 or DMSO in RPMI for 1h at 37°C, washed with ice cold PBS, and lysed in lysis buffer (50mM Tris-HCl pH7,5, 1% NP-40, 1% lauryl maltoside, 165mM NaCl, 10mM EDTA, 10mL NaF, 1mM Na3VO4, 1mM PMSF) for 20min on ice followed by centrifugation for 10min. The soluble fraction was incubated with antibody against Zap70 and ProteinG agarose over night at 4°C. After washing with washing buffer (20mM Tris-HCl pH 7,5, 0,1% NP-40, 0,1% laurly maltoside, 1mM PMSF, 2,5mM NaF. 165mM NaCl), samples were subjected to SDS-PAGE and Western blot. Analyses of immunoprecipitations were performed in two steps. A first SDS-PAGE/western blot, in which 20% of the samples were loaded, was carried out to quantify the amount of Zap70 present in the immunoprecipitations. Subsequently, we performed a second SDS-PAGE/Western blot in which the amount of the loaded wt sample was reduced to normalize the expression of wt Zap70 to that of C575A mutant. This procedure was required to allow a better and reliable quantification of the streptavidin-HRP signal.

### Immunoblot analysis

For immunoblot analysis samples were electrophoresed on SDS polyacrylamide gels and transferred to the polyvinylidene difluoride membrane (Amersham). The membrane was blocked with 5% BSA for 1h, incubated with primary antibodies for 1 h, washed with TBS-T for 20min and TBS for 10min. For detection with ECL system (Amersham Biosciences), membranes were incubated with HRP-labeled secondary antibodies for 1h. For detection with the Odyssey system, membranes were incubated with the respectively labelled secondary antibodies.

### Pulse chase

10^6^ HEK-293T cells were seeded into a well of a 6-well plate coated with poly-D-lysine. The next day, 6μg of Zap70-Myc-DDK or control plasmids were transfected in HEK293T cells. The following day, cells were starved in DMEM lacking cysteine and methionine for 40min followed by incubation with 100μCi of [S35]methionine and cysteine in DMEM lacking cysteine and methionine supplemented with dialyzed FCS for 15min. After washing cells were incubated with normal DMEM/10% FCS for the indicated time points followed by lysis in RIPA buffer (20mM Tris-HCl pH 7,5, 1% Triton X-100, 1mM EDTA, 100mM NaCl, 0,5% sodium deoxycholate, 0,1% SDS, protease inhibitor cocktail (Roche)). Lysates were normalized for the incorporation of radioactive material with [S35] counts in trichloroacetic acid precipitates and subjected to immunoprecipitation using antibody against the FLAG epitope and ProteinA overnight. Beads were washed three times with RIPA buffer and samples were boiled in reducing sample buffer followed by SDS-PAGE and autoradiography.

### Analysis of phosphorylated Erk1/2

P116 cells expressing Zap70-YFP constructs were stimulated with anti-CD3 antibodies immobilized on microspheres as described elsewhere [40]. Briefly, 10^6^ cells were stimulated with 5 × 10^5^ microspheres, coated with 5μg/μL biotinylated anti-CD3 antibody, for the indicated time points at 37°C and 350rpm. The reaction was stopped by addition of ice cold PBS followed by centrifugation. Subsequently, cells were fixed for 10min with 4% PFA. Cells were transferred into cold methanol and stored at -20°C. For intracellular analysis of phosphorylated Er1/2 cells were resuspended in 0,5%BSA in PBS and incubated with anti-pErk1/2 antibody for 1h at RT. Cells were washed with 0,5%BSA in PBS and stained with APC-conjugated goat anti-rabbit secondary antibody. Samples were analyzed on a FACSFortessa (BD Bioscience).

### Statistics

All data are presented as mean ± SEM. Statistical significance was determined between groups using a Student's *t*-test. The minimum acceptable level of significance was *P* < 0.05.

## Abbreviations

Cdc37: Cell division cycle 37
Erk1/2: Extracellular signal-regulated kinases 1/2
Hsp90: Heat shock protein 90
PTK: Protein-tyrosine kinase
TCR: T-cell receptor
Zap70: ζ-chain-associated protein of 70 kDa

## References

[1] Notarangelo LD (2014). Immunodeficiency and immune dysregulation associated with proximal defects of T cell receptor signaling. Current opinion in immunology.

[2] Samelson LE (2011). Immunoreceptor signaling. Cold Spring Harbor perspectives in biology.

[3] Brownlie RJ, Zamoyska R (2013). T cell receptor signalling networks: branched, diversified and bounded. Nature reviews Immunology.

[4] Arpaia E, Shahar M, Dadi H, Cohen A, Roifman CM (1994). Defective T cell receptor signaling and CD8+ thymic selection in humans lacking zap-70 kinase. Cell.

[5] Elder ME, Lin D, Clever J, Chan AC, Hope TJ, Weiss A, Parslow TG (1994). Human severe combined immunodeficiency due to a defect in ZAP-70, a T cell tyrosine kinase. Science.

[6] Roifman CM (1995). A mutation in zap-70 protein tyrosine kinase results in a selective immunodeficiency. Journal of clinical immunology.

[7] Kadlecek TA, van Oers NS, Lefrancois L, Olson S, Finlay D, Chu DH, Connolly K, Killeen N, Weiss A (1998). Differential requirements for ZAP-70 in TCR signaling and T cell development. Journal of immunology.

[8] Negishi I, Motoyama N, Nakayama K, Nakayama K, Senju S, Hatakeyama S, Zhang Q, Chan AC, Loh DY (1995). Essential role for ZAP-70 in both positive and negative selection of thymocytes. Nature.

[9] Katamura K, Tai G, Tachibana T, Yamabe H, Ohmori K, Mayumi M, Matsuda S, Koyasu S, Furusho K (1999). Existence of activated and memory CD4+ T cells in peripheral blood and their skin infiltration in CD8 deficiency. Clinical and experimental immunology.

[10] Matsuda S, Suzuki-Fujimoto T, Minowa A, Ueno H, Katamura K, Koyasu S (1999). Temperature-sensitive ZAP70 mutants degrading through a proteasome-independent pathway. Restoration of a kinase domain mutant by Cdc37. The Journal of biological chemistry.

[11] Chan AY, Punwani D, Kadlecek TA, Cowan MJ, Olson JL, Mathes EF, Sunderam U, Fu SM, Srinivasan R, Kuriyan J, Brenner SE, Weiss A, Puck JM (2016). A novel human autoimmune syndrome caused by combined hypomorphic and activating mutations in ZAP-70. The Journal of experimental medicine.

[12] Carreras J, Villamor N, Colomo L, Moreno C, Ramon y, Cajal S, Crespo M, Tort F, Bosch F, Lopez-Guillermo A, Colomer D, Montserrat E, Campo E (2005). Immunohistochemical analysis of ZAP-70 expression in B-cell lymphoid neoplasms. The Journal of pathology.

[13] Efremov DG, Gobessi S, Longo PG (2007). Signaling pathways activated by antigen-receptor engagement in chronic lymphocytic leukemia B-cells. Autoimmunity reviews.

[14] Wang H, Kadlecek TA, Au-Yeung BB, Goodfellow HE, Hsu LY, Freedman TS, Weiss A (2010). ZAP-70: an essential kinase in T-cell signaling. Cold Spring Harbor perspectives in biology.

[15] Brdicka T, Kadlecek TA, Roose JP, Pastuszak AW, Weiss A (2005). Intramolecular regulatory switch in ZAP-70: analogy with receptor tyrosine kinases. Molecular and cellular biology.

[16] Deindl S, Kadlecek TA, Brdicka T, Cao X, Weiss A, Kuriyan J (2007). Structural basis for the inhibition of tyrosine kinase activity of ZAP-70. Cell.

[17] Au-Yeung BB, Deindl S, Hsu LY, Palacios EH, Levin SE, Kuriyan J, Weiss A (2009). The structure, regulation, and function of ZAP-70. Immunological reviews.

[18] Yan Q, Barros T, Visperas PR, Deindl S, Kadlecek TA, Weiss A, Kuriyan J (2013). Structural basis for activation of ZAP-70 by phosphorylation of the SH2-kinase linker. Molecular and cellular biology.

[19] Yang M, Chen T, Li X, Yu Z, Tang S, Wang C, Gu Y, Liu Y, Xu S, Li W, Zhang X, Wang J, Cao X (2015). K33-linked polyubiquitination of Zap70 by Nrdp1 controls CD8(+) T cell activation. Nature immunology.

[20] Ivanova E, Carpino N (2016). Negative regulation of TCR signaling by ubiquitination of Zap-70 Lys-217. Molecular immunology.

[21] Corcoran A, Cotter TG (2013). Redox regulation of protein kinases. The FEBS journal.

[22] Wood ST, Long DL, Reisz JA, Yammani RR, Burke EA, Klomsiri C, Poole LB, Furdui CM, Loeser RF (2016). Cysteine-Mediated Redox Regulation of Cell Signaling in Chondrocytes Stimulated With Fibronectin Fragments. Arthritis & rheumatology.

[23] Visperas PR, Winger JA, Horton TM, Shah NH, Aum DJ, Tao A, Barros T, Yan Q, Wilson CG, Arkin MR, Weiss A, Kuriyan J (2015). Modification by covalent reaction or oxidation of cysteine residues in the tandem-SH2 domains of ZAP-70 and Syk can block phosphopeptide binding. The Biochemical journal.

[24] Giannoni E, Buricchi F, Raugei G, Ramponi G, Chiarugi P (2005). Intracellular reactive oxygen species activate Src tyrosine kinase during cell adhesion and anchorage-dependent cell growth. Molecular and cellular biology.

[25] Leonberg AK, Chai YC (2007). The functional role of cysteine residues for c-Abl kinase activity. Molecular and cellular biochemistry.

[26] Ushio-Fukai M, Alexander RW, Akers M, Yin Q, Fujio Y, Walsh K, Griendling KK (1999). Reactive oxygen species mediate the activation of Akt/protein kinase B by angiotensin II in vascular smooth muscle cells. The Journal of biological chemistry.

[27] Nakashima I, Takeda K, Kawamoto Y, Okuno Y, Kato M, Suzuki H (2005). Redox control of catalytic activities of membrane-associated protein tyrosine kinases. Archives of biochemistry and biophysics.

[28] Poole LB, Klomsiri C, Knaggs SA, Furdui CM, Nelson KJ, Thomas MJ, Fetrow JS, Daniel LW, King SB (2007). Fluorescent and affinity-based tools to detect cysteine sulfenic acid formation in proteins. Bioconjugate chemistry.

[29] Williams BL, Schreiber KL, Zhang W, Wange RL, Samelson LE, Leibson PJ, Abraham RT (1998). Genetic evidence for differential coupling of Syk family kinases to the T-cell receptor: reconstitution studies in a ZAP-70-deficient Jurkat T-cell line. Molecular and cellular biology.

[30] Senga T, Miyazaki K, Machida K, Iwata H, Matsuda S, Nakashima I, Hamaguchi M (2000). Clustered cysteine residues in the kinase domain of v-Src: critical role for protein stability, cell transformation and sensitivity to herbimycin A. Oncogene.

[31] Veillette A, Dumont S, Fournel M (1993). Conserved cysteine residues are critical for the enzymatic function of the lymphocyte-specific tyrosine protein kinase p56lck. The Journal of biological chemistry.

[32] Li J, Buchner J (2013). Structure, function and regulation of the hsp90 machinery. Biomedical journal.

[33] Kimura Y, Rutherford SL, Miyata Y, Yahara I, Freeman BC, Yue L, Morimoto RI, Lindquist S (1997). Cdc37 is a molecular chaperone with specific functions in signal transduction. Genes & development.

[34] Kato M, Iwashita T, Akhand AA, Liu W, Takeda K, Takeuchi K, Yoshihara M, Hossain K, Wu J, Du J, Oh C, Kawamoto Y, Suzuki H (2000). Molecular mechanism of activation and superactivation of Ret tyrosine kinases by ultraviolet light irradiation. Antioxidants & redox signaling.

[35] Oo ML, Senga T, Thant AA, Amin AR, Huang P, Mon NN, Hamaguchi M (2003). Cysteine residues in the C-terminal lobe of Src: their role in the suppression of the Src kinase. Oncogene.

[36] Rahman MA, Senga T, Oo ML, Hasegawa H, Biswas MH, Mon NN, Huang P, Ito S, Yamamoto T, Hamaguchi M (2008). The cysteine-cluster motif of c-Yes, Lyn and FAK as a suppressive module for the kinases. Oncology reports.

[37] Rahman MA, Senga T, Ito S, Hyodo T, Hasegawa H, Hamaguchi M (2010). S-nitrosylation at cysteine 498 of c-Src tyrosine kinase regulates nitric oxide-mediated cell invasion. The Journal of biological chemistry.

[38] Trevillyan JM, Chiou XG, Ballaron SJ, Tang QM, Buko A, Sheets MP, Smith ML, Putman CB, Wiedeman P, Tu N, Madar D, Smith HT, Gubbins EJ (1999). Inhibition of p56(lck) tyrosine kinase by isothiazolones. Archives of biochemistry and biophysics.

[39] Lee JW, Kim JE, Park EJ, Kim JH, Lee CH, Lee SR, Kwon J (2004). Two conserved cysteine residues are critical for the enzymic function of the human platelet-derived growth factor receptor-beta: evidence for different roles of Cys-822 and Cys-940 in the kinase activity. The Biochemical journal.

[40] Arndt B, Poltorak M, Kowtharapu BS, Reichardt P, Philipsen L, Lindquist JA, Schraven B, Simeoni L (2013). Analysis of TCR activation kinetics in primary human T cells upon focal or soluble stimulation. Journal of immunological methods.

